# *Chrysosphaerella septentrionalis* sp. nov. (Chrysophyceae, Chromulinales), a New Species from the Arctic Including the Description of *Chrysosphaerellaceae*, fam. nov.

**DOI:** 10.3390/plants11223166

**Published:** 2022-11-18

**Authors:** Dmitry Kapustin, Maxim Kulikovskiy

**Affiliations:** Timiryazev Institute of Plant Physiology, Russian Academy of Sciences, Botanicheskaya Street 35, 127276 Moscow, Russia

**Keywords:** chrysophytes, siliceous scales, morphology, ultrastructure, SEM, hidden diversity, pseudocryptic taxa, Russia

## Abstract

A new species, *Chrysosphaerella septentrionalis*, is described from a peat bog located on the bank of the Paz River (Pasvik Nature Reserve, Murmansk Region, Russia). Scale ultrastructure was studied using a scanning electron microscope. Morphologically, *C. septentrionalis* seems to closely resemble *C. longispina*. However, *C. septentrionalis* possesses subcircular scales in addition to the oval scales, and they are much smaller than in *C. longispina*. We suppose that *C. septentrionalis* is the first pseudocryptic species within the *C. longispina* complex. Additionally, we proposed an infrageneric classification of *Chrysosphaerella* based on the scale structure and divided the genus into three sections: *Chrysosphaerella*, *Brevispinae* sect. nov. and *Septispinae* sect. nov. The formal description of a new family *Chrysosphaerellaceae* fam. nov. is also provided.

## 1. Introduction

The genus *Chrysosphaerella*—with a single species, *C. longispina*—was described by Lauterborn [1]. Its spherical colonies have a *Synura*-like appearance but possess long spines. Several years later, he provided additional observations and a figure of this species [2].

Korshikov [3] described a second species within the genus, *Chrysosphaerella brevispina*. He carefully illustrated the scales and spines of both species and showed the difference in scale ornamentation and spine length. Moreover, he discovered a second short flagellum in both *C. longispina* and *C. brevispina* and proposed to place *Chrysosphaerella* within the family Synuraceae.

The first electron microscopical observations on the ultrastructure of *Chrysosphaerella* scales were made by Fott and Ludvík [4] who studied *Chrysosphaerella brevispina*. Later, Harris and Bradley [5] amended the description of this species and synonymized *C. rodhei* Skuja [6] with it. In 1964, Bradley—based on light and electron microscopical observations–described a new species, *C. multispina* [7]. This species resembled *C. longispina* but differed mainly in having a greater number of spines of different length. At the time of description of *C. multispina*, the scale ultrastructure of *C. longispina* was unknown, so Bradley [7] made a comparison based on Korshikov’s light microscopical observations. According to Bradley, the scales of *C. multispina* have a smooth margin and reticulate pattern at the center, whereas in *C. longispina,* scales have a “thickened inner ring with spokes radiating to margin” ([7], p. 331). Subsequently, many authors pointed out that *C. longispina* and *C. multispina* might be conspecific [8,9,10,11]. Finally, Nicholls [12] amended *C. longispina* and regarded *C. multispina* as its synonym.

Almost simultaneously, Wujek et al. [13] and Preisig and Takahashi [14] described the same species under the names *Chrysosphaerella coronacircumspina* and *C. solitaria,* respectively. Unlike *C. longispina*—the generitype—which is a colonial organism, this species is unicellular. Preisig and Takahashi [14] even established a new subgenus, *Chrysosphaerella* subgen. *Pseudochrysosphaerella,* to accommodate a solitary species of *Chrysosphaerella*. However, this taxon no longer exists because its type species, *Chrysosphaerella salina* Birch-Andersen is not a chrysophyte, and it has been transferred to *Thaumatomastix* [15].

After the description of a noncolonial species, the differences between *Chrysosphaerella* and *Spiniferomonas*, which has similar scale ultrastructure but is solitary rather than colonial [16], became unclear. Nicholls [12] proposed that both genera could be distinguished by the structure of spine-scales: in *Spiniferomonas* the base of the spine is cone-shaped, cup-shaped, or the spine is fixed to a simple flat disc, whereas in *Chrysosphaerella,* the base of the spine is bobbin- or pulley-like. However, after the discovery of two new species which had scale morphological features common to both *Spiniferomonas* and *Chrysosphaerella,* he changed his mind and decided to separate these genera based on cell habit—colonial in *Chrysosphaerella* or solitary in *Spiniferomonas* [17]. Kristiansen and Tong [18] disagreed with him and argued that morphology of siliceous structures has more taxonomic value than cell habit. The latter point of view is currently widely accepted; however, no additional data are available to support any of these hypotheses.

Taxonomic placement of *Chrysosphaerella* remained unclear. It was included together with other photosynthetic (*Spiniferomonas* and *Polylepidomonas*) and heterotrophic (*Paraphysomonas* sensu lato) genera in the family Paraphysomonadaceae [19]. Molecular analysis performed by Škaloud et al. [20] clearly showed that *Chrysosphaerella* is unrelated to *Paraphysomonas*, so, *Chrysosphaerella* cannot belong to the Paraphysomonadaceae, which currently include only the heterotrophic genus *Paraphysomonas* sensu stricto [21]. Recently, Kapustin et al. [22] have proposed a provisional family name Chrysosphaerellaceae to classify *Chrysosphaerella* within the Chromulinales. Therefore, we aimed to describe a new species of *Chrysosphaerella*—*C. septentrionalis*, belonging to the *C. longispina* complex—and formally describe a new family, Chrysosphaerellaceae.

## 2. Results

**Figure 1:**
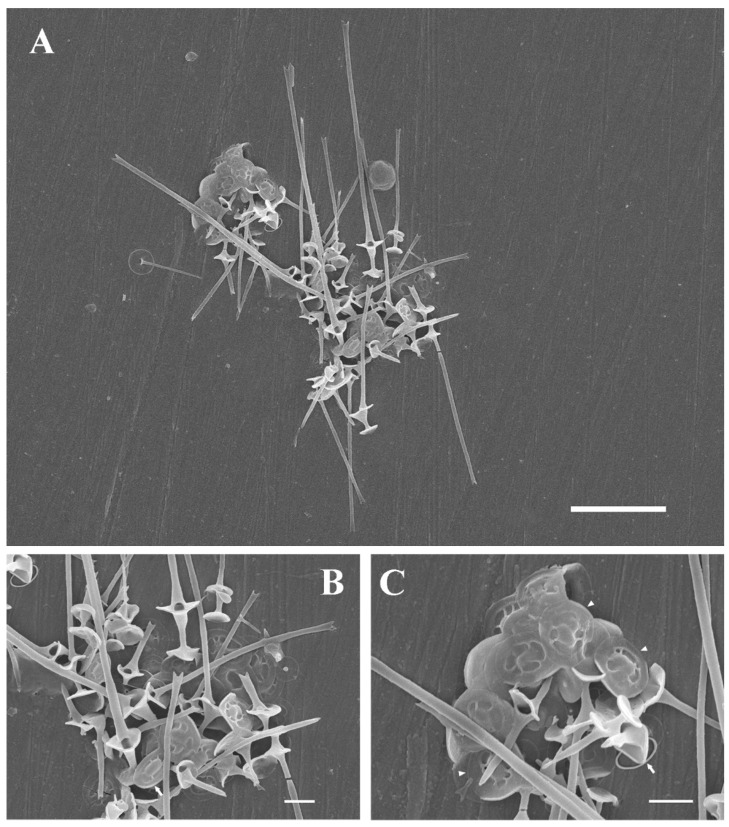
(**A**–**C**) *Chrysosphaerella septentrionalis* sp. nov., SEM. (**A**) General view; (**B**) close-up view of the scales. Note the oval plate-like scale; (**C**) close-up view of the subcircular (arrowhead) and the oval unpatterned scales (arrow). Scale bars: (**A**): 10 µm; (**B**,**C**): 2 µm.

***Chrysosphaerella septentrionalis*** Kapustin **sp. nov.** (Figure 1).

Colonies multicellular, dimensions unknown. Cells covered with plate-like and spine-like siliceous scales. Plate-like scales oval to subcircular, 2.4–3.0 × 1.8–3.0 µm, consist of a raised and smooth central area (cupola), a plain smooth marginal rim and radial ribs between them; small oval unpatterned scales (2.1 × 1.1 µm) also occur. Spine-scales (4.3–8.3 µm and 13.0–31.3 µm in length) consist of two baseplates connected by a wineglass-shaped shaft and a tubular spine with a flattened and bifurcate tip; a large circular hole presents at the spine base. Stomatocysts unknown.

**Holotype** (here designated): Portion of a single gathering of cells on SEM stub #P32 deposited at the Herbarium of the Papanin Institute for Biology of Inland Waters RAS, Borok (IBIW). Sample was collected on 19 June 2019. Figure 1A illustrates the holotype.

**Type Locality**: Peat bog on the bank of the Paz River (69°23.489′ N, 29°45.388′ E), Pasvik Nature Reserve, Murmansk Region (Russia).

**Etymology**: The species epithet, which means “northern” in Latin, refers to the distribution of this species in a high-latitude region.

**Distribution**: So far, this species is known from its type locality only.

## 3. Discussion

### 3.1. Species Diversity of Chrysosphaerella

Like in the case of other silica-scaled chrysophytes, the taxonomy of the genus *Chryso-sphaerella* is based almost exclusively on the ultrastructure of spines and scales. With the addition of our new species, there are 12 currently accepted taxa within the genus (Table 1). Molecular data are available for three species, namely *C. brevispina*, *C. longispina*, and *C. rotundata.*

**Figure 2:**
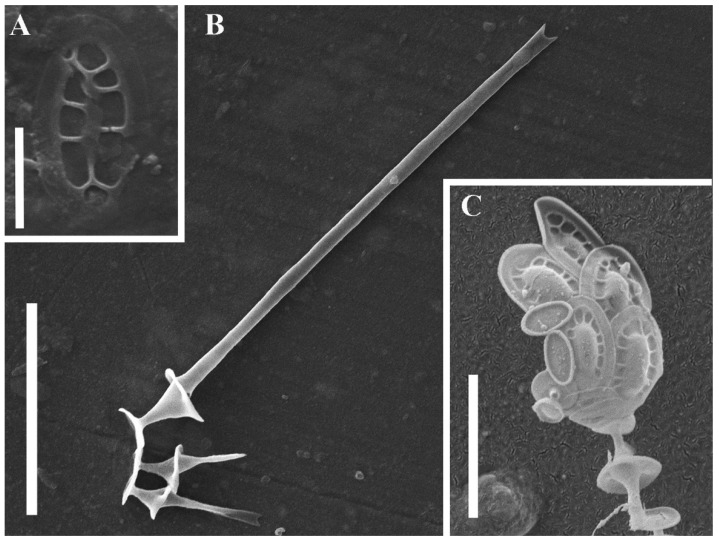
(**A**–**C**) *Chrysosphaerella longispina* from the Moscow Region, SEM. (**A**) Oval plate-like scale; (**B**) spine-like scales of different size classes; (**C**) a part of the cell armor consisting of the patterned and unpatterned oval plate-like scales and the spine-like scale. Scale bars: (**A**): 2 µm; (**B**): 10 µm; (**C**): 5 µm.

Our new species, *Chrysosphaerella septentrionalis,* is extremely similar to *C. longispina* (Figure 2), the generitype, and *C. multispina*. The latter taxon is considered to be conspecific to *C. longispina* [12]. Additionally, in terms of botanical nomenclature, *C. multispina* is invalid because the type has not been indicated (Art. 40.1).

In contrast to *C. longispina* which has elliptical or oval plate-scales up to 6 µm long, the majority of plate-scales in *C. septentrionalis* are subcircular and do not exceed 3 µm in diameter. The scale structure in both species is similar (see Figure 1 and Figure 2). It consists of a raised and smooth central area (cupola) connected by radial ribs to a smooth marginal rim [12,31]. Interestingly, those subcircular or broadly elliptic plate-like scales were depicted in micrographs published by Bradley (Pl. 6, Figure 44, [7]) and Asmund (Figure 5, [10]) for *C. multispina* or Siver et al. (Figure 7H, [32]) for *C. longispina*. However, unlike *C. septentrionalis* in the abovementioned cases, the circular scales are not a dominant scale type and, their size does not exceed the size of elliptic scales.

Additionally, *C. septentrionalis* has spine-scales of two size classes, similarly to *C. longispina*. However, in *C. longispina,* they have a wider range of length [12,31]. Morphometrical data of both species are summarized in Table 2.

Relatively recently, Škaloudová and Škaloud [28] clearly showed the existence of hidden diversity within the genus *Chrysosphaerella* and described the first pseudocryptic species, *C. rotundata* which can be attributed to the *C. brevispina*-species complex. *Chrysosphaerella septentrionalis* belongs to another species complex, namely *C. longispina.* Probably, the specimen depicted by Bessudova et al. [33] and identified as *C. longispina* represents a currently undescribed species from this species complex. Therefore, we totally agree with Němcová et al. [34], who stated that the diversity of *Chrysosphaerella* is largely undescribed, and our discovery of a new species *C. septentrionalis* supports this view.

### 3.2. Infrageneric Classification of the Genus Chrysosphaerella

There has only been one attempt to develop an infrageneric classification of *Chrysosphaerella* based on colonial vs. solitary habitat [14]. Unfortunately, the type of the subgenus *Pseudochrysosphaerella*, which comprises solitary species, belongs to the thaumatomonads (Cercozoa) rather than the chrysophytes. Thus, this name cannot be used anymore.

In our opinion, based on the scale ultrastructure the genus can be divided into the following three sections: *Chrysosphaerella*, *Brevispinae,* and *Septispinae*.

1. ***Chrysosphaerella*** sect. ***Chrysosphaerella***

Exclusively colonial chrysophytes. Plate-scales consist of a raised and smooth central area (cupola) connected by radial ribs to a smooth marginal rim. Spine-scales consist of two baseplates and a spine with a large hole at its base.

**Type species**: *Chrysosphaerella longispina* Lauterborn. 1896. Zool. Anz. 19: 16.

At present, this section consists of two species: *C. longispina* and *C. septentrionalis*.

2. ***Chrysosphaerella*** sect. ***Brevispinae*** Kapustin, **sect. nov.**

Colonial and solitary chrysophytes. Plate-scales with a thickened oval ring in the central part on the exterior surface and ornamented with a scalloped oval shaped pattern on the undersurface. Spine-scales consist of two baseplates and a spine with a large hole at its base.

**Type species** (here designated): *Chrysosphaerella brevispina* Korshikov. 1941. Arch. Protistenk. 95: 31, 32, Figure 7.

At present, this section includes eight taxa: *C. astrea*, *C. baikalensis*, *C. brevispina*, *C. coronacircumspina* var. *coronacircumspina*, *C. coronacircumspina* var. *grandibasa*, *C. enigmata*, *C. nichollsii,* and *C. rotundata*.

3. ***Chrysosphaerella*** sect. ***Septispinae*** Kapustin, **sect. nov.**

Exclusively solitary chrysophytes. Plate-scales with more or less oval rings with crenulated margins, or with 10–15 min crenulated annular structures. Spine-scales consist of a single baseplate separated from the spine by a septum. A circular hole in the spine wall is located at various distances above the septum.

**Type species** (here designated): *Chrysosphaerella septispina* (K.H. Nicholls) Kristiansen and D. Tong. 1989. Nord. J. Bot. 9: 331. (≡*Spiniferomonas septispina* K.H. Nicholls. 1984. Pl. Syst. Evol. 148: 104, 105, Figures 1–5).

At present, this section consists of two species: *C. septispina* and *C. annulata*.

### 3.3. Taxonomic Placement of the Genus Chrysosphaerella

The views on the taxonomic placement of *Chrysosphaerella* have changed drastically for over the last 120 years. Lemmermann [35] placed *Chrysosphaerella* in the Mallomonadaceae, within the order Phaeozoosporinae. Interestingly, *Actinoglena klebsiana* Zacharias, which is now considered to be conspecific with *C. longispina*, was placed by him in the Synuraceae under the name *Synura klebsiana* (Zacharias) Lemmermann. Later, Pascher [36] classified *Chrysosphaerella* within the family Mallomonadaceae, in the order Chromulinales.

It should be noted that the number of visible flagella and their length was considered as an important taxonomic character at the ordinal level [37,38]. Pascher recognized three orders: Chromulinales (one flagellum), Isochrysidales (two equal flagella), and Ochromonadales (two unequal flagella). Therefore, when Korshikov [3] discovered a second short flagellum in both *C. brevispina* and *C. longispina*, he proposed to transfer *Chrysosphaerella* to the family Synuraceae within the order Ochromonadales. This point of view was accepted in famous treatments on chrysophytes by Bourrelly [39] and Starmach [40].

Preisig and Hibberd [19] showed that cell ultrastructure of the members from the genera *Chrysosphaerella*, *Paraphysomonas*, *Spiniferomonas,* and *Polylepidomonas* is much more similar to that of *Ochromonas* and *Chromulina* than to that of *Mallomonas* and *Synura.* Therefore, they decided to establish a new family, Paraphysomonadaceae, to accommodate *Chrysosphaerella*, *Paraphysomonas*, *Spiniferomonas,* and *Polylepidomonas*.

Cavalier-Smith et al. [41] erected the order Paraphysomonadales for exclusively colorless chrysophytes. In several studies, it was shown that *Paraphysomonas* sensu lato formed a distinct lineage which took a basal position to all other chrysophytes [20,42,43].

The phylogenetic position of *Chrysosphaerella* was reported by Andersen [44] based on a single unidentified colony. This isolate was closely related to the nonscaled genera *Chromulina*, *Chrysamoeba,* and *Oikomonas.* Subsequently, Škaloud et al. [20] corroborated this phylogenetic position of *Chrysosphaerella* by adding SSU rDNA and *rbc*L sequences from cultured *Chrysosphaerella* taxa. Therefore, they clearly showed that *Chrysosphaerella* is unrelated to *Paraphysomonas*. Currently, the family Paraphysomonadaceae is restricted to a single genus, *Paraphysomonas* sensu stricto, and together with another monotypic family, Lepidochromonadaceae (=Clathromonadidae), they form the order Paraphysomonadales [21,45].

Although Kapustin et al. [22] used the provisional family name Chrysosphaerellaceae, they did not provide its description. It should be noted that the name Chrysosphaerellaceae in Pascher [46] is a misprint of Chrysosphaeraceae. Therefore, a new family is formally described below:

**Chrysosphaerellaceae** Kapustin, **fam. nov**.

Colonial or solitary photosynthetic chrysophytes. Flagella two unequal. Chloroplasts one or two yellow-brown. Cells covered with siliceous scales of two main types, plate-like and spine-like. Plate-like scales elliptical, oval, or subcircular. Spine-like scales consist of a single or two base-plates and flat or tubular spine. Two genera: *Chrysosphaerella* and *Spiniferomonas* (=*Chromophysomonas* Preisig & Hibberd).

**Type genus** (here designated): *Chrysosphaerella* Lauterborn.

Although the members of *Spiniferomonas* remain unsequenced, we tentatively place this genus in the Chrysosphaerellaceae based on the similarities in scale structure. The genus *Polylepidomonas* most likely requires its own separate family.

## 4. Materials and Methods

A sample containing a putatively new species of *Chrysosphaerella* was collected from a peat bog located on the bank of the Paz River (69°23.489′ N, 29°45.388′ E), Pasvik Nature Reserve, Murmansk Region (Russia) by squeezing water from *Sphagnum* on 19 June 2019. A sample containing *C. longispina* was collected from the surface water layer of the Marfino bog (56°04′10.2″ N 37°32′31.8″ E), Moscow Region (Russia) using a 20 µm mesh plankton net on 14 May 2022. Environmental variables were not measured.

For scanning electron microscope (SEM) studies, a few drops from the unfixed samples were placed on aluminum stubs, air-dried, and sputter-coated with gold for 10 min. Observations were carried out with JEOL 6510 LV (IBIW RAS) or TESCAN Vega III (PIN RAS) scanning electron microscopes.

## Figures and Tables

**Table 1:** Checklist of all previously described species of the genus *Chrysosphaerella*.

Taxon	Taxonomic Status	Reference(s)
*Chrysosphaerella annulata* Kristiansen & D. Tong	Accepted	[18]
*Chrysosphaerella astrea* Dürrschmidt	Accepted	[23]
*Chrysosphaerella baikalensis* Popovskaya	Accepted (Probably, it is conspecific with *C. brevispina*)	[24]
*Chrysosphaerella brevispina* Korshikov	Accepted	[5]
*Chrysosphaerella conradii* Bourrelly	Synonym of *C. brevispina*	[10]
*Chrysosphaerella coronacircumspina* Wujek & Kristiansen var. *coronacircumspina*	Accepted	[13]
*Chrysosphaerella coronacircumspina* var. *grandibasa* Balonov	Accepted	[25]
*Chrysosphaerella enigmata* (K.H. Nicholls) Kristiansen & D. Tong	Accepted	[18]
*Chrysosphaerella longispina* Lauterborn	Accepted	[12]
*Chrysosphaerella multispina* Bradley	Invalid Synonym of *C. longispina*	[12]
*Chrysosphaerella nichollsii* D. Kapustin & E.S. Gusev	Accepted	[26]
*Chrysosphaerella parva* Asmund	Synonym of two *Spiniferomonas* taxa, *S. abei* E. Takahashi and *S. bilacunosa* E. Takahashi	[27]
*Chrysosphaerella patelliformis* E. Takahashi & Hara	Synonym of *Thaumatomastix patelliformis* (E. Takahashi & Hara) Beech & Moestrup	[15]
*Chrysosphaerella rodhei* Skuja	Synonym of *C. brevispina*	[10]
*Chrysosphaerella rotundata* Škaloudová & Škaloud	Accepted	[28]
*Chrysosphaerella salina* Birch-Andersen	Synonym of *Thaumatomastix salina* (Birch-Andersen) P.L. Beech & Moestrup	[15]
*Chrysosphaerella septispina* (K.H. Nicholls) Kristiansen & D. Tong	Accepted	[18]
*Chrysosphaerella setifera* Schiller	Insufficiently described	[10]
*Chrysosphaerella solitaria* Preisig & E. Takahashi	Synonym of *C. coronacircumspina*	[29]
*Chrysosphaerella triangulata* Balonov	Synonym of *Thaumatomastix triangulata* (Balonov) P.L. Beech & Moestrup emend. K.H. Nicholls	[15,30]
*Chrysosphaerella tripus* E. Takahashi & Hara	Synonym of *Thaumatomastix tripus* (E. Takahashi & Hara) P.L. Beech & Moestrup	[15]

**Table 2:** Morphometrical comparison between *Chrysosphaerella septentrionalis* and *C. longispina* (incl. *C. multispina*).

Species	Plate-like Scales (Length × Width, µm)	Spine-like Scales (Length, µm)	Reference
*Chrysosphaerella septentrionalis*	2.4–3.0 × 1.8–3.0 unpatterned scales: 2.1 × 1.1	4.3–8.3 13.0–31.3	This study
*Chrysosphaerella longispina* (=*C. multispina*)	0.6–6.0	5–10 20–25 35–40	[7]
	2.0–2.5 × 1.25–1.6 4.6–6.0 × 2.1–3.3 unpatterned scales: 1.3–1.7 × 0.9–1.0	3–4 up to 50	[8]
	3.5–6.0 × 2.2–3.0 unpatterned scales: 1.8–2.5 × 1.0–1.6	3–85	[12]
	4.2–2.1 (mean size)	3.7–6.8 13–53 up to 71	[31]

## Data Availability

Not applicable.
